# Accumulating Research: A Systematic Account of How Cumulative Meta-Analyses Would Have Provided Knowledge, Improved Health, Reduced Harm and Saved Resources

**DOI:** 10.1371/journal.pone.0102670

**Published:** 2014-07-28

**Authors:** Mike Clarke, Anne Brice, Iain Chalmers

**Affiliations:** 1 All-Ireland Hub for Trials Methodology Research, Queen's University Belfast, Belfast, United Kingdom; 2 James Lind Initiative, Oxford, United Kingdom; Copenhagen University Hospital Gentofte, Denmark

## Abstract

**Background:**

“Cumulative meta-analysis” describes a statistical procedure to calculate, retrospectively, summary estimates from the results of similar trials every time the results of a further trial in the series had become available. In the early 1990s, comparisons of cumulative meta-analyses of treatments for myocardial infarction with advice promulgated through medical textbooks showed that research had continued long after robust estimates of treatment effects had accumulated, and that medical textbooks had overlooked strong, existing evidence from trials. Cumulative meta-analyses have subsequently been used to assess what could have been known had new studies been informed by systematic reviews of relevant existing evidence and how waste might have been reduced.

**Methods and Findings:**

We used a systematic approach to identify and summarise the findings of cumulative meta-analyses of studies of the effects of clinical interventions, published from 1992 to 2012. Searches were done of PubMed, MEDLINE, EMBASE, the Cochrane Methodology Register and Science Citation Index. A total of 50 eligible reports were identified, including more than 1,500 cumulative meta-analyses. A variety of themes are illustrated with specific examples. The studies showed that initially positive results became null or negative in meta-analyses as more trials were done; that early null or negative results were over-turned; that stable results (beneficial, harmful and neutral) would have been seen had a meta-analysis been done before the new trial; and that additional trials had been much too small to resolve the remaining uncertainties.

**Conclusions:**

This large, unique collection of cumulative meta-analyses highlights how a review of the existing evidence might have helped researchers, practitioners, patients and funders make more informed decisions and choices about new trials over decades of research. This would have led to earlier uptake of effective interventions in practice, less exposure of trial participants to less effective treatments, and reduced waste resulting from unjustified research.

## Introduction

In 1992, a team lead by Tom Chalmers and Fred Mosteller introduced the term ‘cumulative meta-analysis' to describe a statistical procedure to calculate, retrospectively, summary estimates based on the results of similar trials every time the results of a further trial in the series had become available [1]. One of their two papers published in 1992 made clear how important this procedure was for auditing both research and healthcare advice. Comparisons of the results of cumulative meta-analyses of treatments for myocardial infarction with the advice that had been promulgated through medical textbooks [2] made clear not only that research had continued long after robust estimates of treatment effects had accumulated, but also that medical textbooks had overlooked strong, existing evidence from clinical trials, both of beneficial and of lethal effects of treatments [3].

Cumulative meta-analyses have subsequently been used to assess what could have been known had the design of new studies been informed by reference to systematic reviews of relevant existing evidence and how these might have reduced waste [4]. Cumulative meta-analyses emphasise the need for the design of new studies to be informed by existing research [5] and for the results of new studies to be set in the context of updated systematic reviews of the relevant evidence from all sufficiently similar studies [6]. The idea of using the accumulating evidence to make decisions about the design and ongoing conduct of trials is not new: the report of a case study published by Henderson and colleagues nearly 20 years ago noted “Our thesis is that if related published trials are available, a meta-analysis should be started in the planning stages of a clinical trial, continued through the ongoing conduct of the trial, and performed as one analysis among many in the final analysis of the trial” [7]. Such reviews and meta-analyses help to provide the ethical, scientific and environmental justification for both new study and for any future studies [8].

In this methodological review, we use systematic methods to search for and summarise the findings of cumulative meta-analyses of studies of the effects of clinical interventions, published from 1992 to 2012. We describe the different settings for these studies and explore their findings in the context of unnecessary duplication of effort or waste if trials were done after a robust finding would have been found if a review and meta-analysis of existing research had been performed. By conducting this research as a systematic review, our aim is to provide the most comprehensive collection of cumulative meta-analysis of studies of healthcare interventions. The searching for this review also identified several cumulative meta-analyses in other types of health research, which are not summarised here but have been discussed in brief elsewhere [4]. For example, if epidemiological studies investigating possible aetiological factors in sudden infant death syndrome had taken proper account of the accumulating evidence, the lethal effect of ‘front lying’ would have been recognized at least a decade earlier, and tens of thousands of infant deaths could have been avoided [9]. A cumulative meta-analysis of 55 studies that continued to be conducted over more than two decades showed that for over 17 years there had been ample evidence that never-smoking women who had been exposed to spousal smoking were more likely than controls to develop lung cancer [10].

## Methods

### Inclusion criteria

Studies were eligible if they included a cumulative meta-analysis of studies of the relative effects of alternative healthcare interventions. Ideally, the cumulative meta-analysis would be presented as a graph showing the summary estimates as each study's result was added to the meta-analyses in the order this had been published or became available in some other way. However, studies were eligible if these graphs or the separate summary estimates were not available, but the general findings or implications of the cumulative meta-analyses were reported. Studies were eligible if a single cumulative meta-analysis was presented, or if a meta-epidemiological project had been done in which numerous systematic reviews or meta-analyses were included. Eligible cumulative meta-analyses had sometimes been done as part of the planning process for a new study to explore how the evidence base had evolved. Studies were not included if only surrogate outcome measures were used unless - like blood pressure and severe anaemia, for example - these were unambiguously important. The searches also identified cumulative meta-analysis in other types of health research (including observational epidemiology and genetics) but these were not eligible for this review.

### Search strategy

We wished to identify published reports containing graphs of cumulative meta-analyses. An initial search of Pubmed Clinical Queries using the term ‘cumulative meta-analysis’ retrieved 822 records. The selected papers were screened by one author (IC), who selected a set of 23 records which were added to a set of core articles that had been previously identified as part of this author's general interest in this area. This full set of papers were then analysed to obtain ideas for free-text search terms and index or MeSH terms for use in a final search strategy.

Searches were then run on the following databases: MEDLINE, OVID (1946–2012, In-Process and other non-indexed citations); EMBASE, OVID (1947–2012); the Cochrane Methodology Register (2012); and Science Citation Index (2012). No language or publication date restrictions were applied. The following search strategy was used for MEDLINE: cumulative adj10 meta?analys$.mp. OR (cumulative.mp. and meta-analysis.pt.). The search of the Cochrane Methodology Register combined the output of a simple search for the word ‘cumulative’ in the abstract with that for a search for records that had been assigned the term ‘Meta-analysis updating and cumulative meta-analysis’ by the compilers of the Register. The searches were conducted in June 2012.

The reference lists of relevant systematic reviews and other publications were checked to identify additional articles [11]. A search was undertaken in Google Scholar to identify relevant terms in the full text of articles where relevant concepts had not been located in bibliographic databases using searches of titles, abstracts and index terms.

### Assessment of studies, data extraction and analysis

Records were exported from Endnote to Sente for first screening by one of the authors (AB). This screening was deliberately over-inclusive, and all potentially eligible articles or unclear articles were checked by a second author (IC). The eligibility of all studies arising from this process was then confirmed by a third author (MC) before their inclusion in this review.

The process of data extraction was piloted in ten records and emerging themes identified. A final data extraction form was then agreed. All eligible papers were read, and passages of text commenting on the details of the cumulative meta-analysis and the implications of the completed cumulative meta-analyses were identified and extracted. This was done by one author (AB) and the data extracted were checked against the original articles by the other authors (IC and MC).

No attempts were made to combine the results of the included studies, because the aim of this review is to present a range of examples and identify themes across the cumulative meta-analyses identified, rather than to attempt to generate a statistical finding. Similarly, this review does not seek to estimate the incidence or prevalence of trials that have been conducted unnecessarily, since that would be done better with a comprehensive study of large cohorts of trials addressing the same or similar questions. Instead, it seeks to identify and report a range of examples to examine whether cumulative meta-analyses might have led to different choices in the conduct of new research.

## Results

### Results of the search

This initial screening identified 942 records needing inspection, of which 818 were excluded in the initial screening. This screening identified 46 records for inclusion and 78 needing further review. The second screening step excluded 74 of these, leaving 50 reports, including more than 1500 cumulative meta-analyses of clinical intervention studies, as eligible.

### Included studies

The included studies were published from 1992 to 2012 inclusive. Most of these studies focused on a single question for a systematic review or meta-analysis, and included just one graphical presentation of a cumulative meta-analysis. The 50 studies addressed a wide range of health problems. Fourteen covered aspects of surgery for a variety of problems (reducing infection and bleeding and improving surgical technique). Eleven studies concerned heart disease, and there were at least two for each of stroke, neonatal problems, infectious disease, cancer, mental health, dental health and respiratory illness. There were single studies in obstetrics, musculoskeletal disease, kidney disease and gastrointestinal disease. The number of individual research studies in each of the cumulative meta-analyses we identified ranged from 4 trials of continuous versus interrupted techniques for elective midline abdominal fascial closure [12] to more than 60 trials for aprotinin in cardiac surgery [13].

Nearly all of the cumulative meta-analyses had been conducted as retrospective exercises to explore how the evidence base had evolved. However, some were used to inform the design of a new study. For example, Algra and van Gijn presented a cumulative meta-analyses which was used to inform the need for the European and Australian Stroke Prevention in Reversible Ischaemia Trial (ESPRIT) [14].

A variety of themes were apparent from the cumulative meta-analyses and these are illustrated here with specific examples. A detailed explanation is not provided for each cumulative meta-analyses, but the citations for all the included studies are available in Appendix S1. The included studies showed that initially positive results became null or negative in the meta-analysis as more trials were done; that early null or negative results were over-turned; that stable results (beneficial, harmful and neutral) would have been seen had a meta-analysis of existing evidence been done before the new trial; and that additional trials had been much too small to resolve the remaining uncertainties.

### Positive results becoming less so

Cumulative meta-analyses have shown how replications have challenged initially favourable results [15]–[18] where the early trials were favourable but not statistically significant. For example, Klein and colleagues examined the effects of cognitive behavioural therapy (CBT) for adolescent depression, using the publication dates for 11 randomised trials to build a cumulative meta-analysis. They found that the effect size from the meta-analyses had decreased steadily from the large effects observed in the earliest trials, with narrowing confidence intervals as the data accumulated. However, they also noted that the trend could be related to methodological differences between the studies and wrote “these differences appear to reflect both a shift from an initial emphasis on demonstrating the efficacy of treatment in controlled research settings to an emphasis on demonstrating the effectiveness of treatment and the application of increased statistical and methodological rigor over time”. They concluded that “the results indicate that CBT may be effective for the acute treatment of depression among adolescents, although treatment effects may be more modest in clinical settings than findings from early trials would suggest” [17].

### Null or negative results becoming positive

Cumulative meta-analyses have also shown how replications have sometimes challenged initially unfavourable results [19]–[20], but these examples are fewer and weaker than for the previous theme. For example, in a systematic review comparing plating versus intramedullary nailing of humeral shaft fractures in adults, the cumulative meta-analyses by Li and colleagues showed that intramedullary nailing might increase the re-operation rate in studies conducted before 2000 (odds radio (OR): 0.39, 95% confidence interval (CI): 0.17 to 0.90, P = 0.03). They noted that although the point estimate was still unfavourable, the difference was not statistically significant in studies conducted after that year (OR: 0.54, 95% CI: 0.27 to 1.08, P = 0.08) [19].

### Stable results

This collection of cumulative meta-analyses includes many that show that a systematic review of existing research would have reduced uncertainty about an intervention. They showed how morbidity and mortality might have been reduced, both for patients in general and for participants in trials in which they may have continued to be allocated to a placebo or a control group when the intervention could have been shown to be effective in a meta-analysis [1]–[2], [12]–[14], [18], [21]–[38].This observation also has implications for waste in research, since new studies might have been regarded as unjustified if a systematic review and meta-analysis had been done as part of its design phase. Two examples, through two decades, are used to illustrate this.

In 1992, Lau and colleagues reported a collection of cumulative meta-analyses of clinical trials that evaluated 15 treatments and preventive measures for acute myocardial infarction. They found a consistent, statistically significant reduction in total mortality for the use of streptokinase (OR: 0.74, 95% CI: 0.59 to 0.92) would have been shown in 1973, after only eight trials involving 2432 patients had been completed. The results of 25 subsequent trials, which enrolled an additional 34,542 patients through 1988, had little or no effect on the odds ratio establishing efficacy, but narrowed the 95% confidence interval [1].

The cumulative meta-analysis by Fergusson and colleagues of aprotinin in cardiac surgery found a dramatically beneficial result in the first trial of 22 patients in 1987 (OR for perioperative blood transfusion: 0.03, 95% CI: 0.00 to 0.56), with the point estimate declining over subsequent randomised trials. By the twelfth study (published in June 1992), the cumulative effect estimate for the odds ratio had stabilized (OR: 0.25, P<0.000001) with a total of approximately 2500 patients randomised. Throughout the cumulative meta-analysis, the upper limit of the confidence interval did not go higher than 0.65, and in this study, the final meta-analyses was 0.34 (95% CI: 0.24–0.41) after the publication of a trial in June 2002, by which time a total of more than 8000 patients had been randomised [13].

### New trials too small to resolve remaining uncertainties

Although systematic reviews of existing evidence sometimes reveal data that are sufficient to answer research questions, cumulative meta-analyses, like that of tranexamic acid [39], have also exposed questions that remain unanswered, but continue to be addressed in studies that are much too small to resolve the remaining uncertainties [15], [40]–[56]. The importance of addressing uncertainties was revealed when a systematic review showed that a series of 16 under-powered trials conducted over 23 years failed to indicate whether steroids given to patients with traumatic brain injury reduced or increased their chances of survival. The uncertainty was only resolved when a very large trial showed reliably that the treatment increased mortality [57].

## Discussion

This large collection of cumulative meta-analyses, from across health and social care, and covering a wide range of research into the effects of interventions, highlights how a review of the existing evidence might have helped researchers, practitioners, patients and funders make more informed decisions and choices about new clinical trials over many decades of research. The importance of taking account of earlier research is not a new concept for either the design or interpretation of new studies. For example, it was highlighted to the British Association for the Advancement of Science by Lord Rayleigh in the nineteenth century [58]–[59].

The cumulative meta-analyses that have been considered together for the first time in this review provide cautionary tales about how new research might have been designed and implemented, when the existing research would have shown it to be unnecessary. Other research, taking a different approach to this review, has also highlighted this problem. In 2011, Herbison and colleagues reported cumulative meta-analysis of data from 65 meta-analyses from 18 Cochrane Reviews. They found that it took a median of four (interquartile range: 1 to 6) studies to get within 10% of the final point estimate. However, they noted that although their study suggests that, in many cases, only a few trials are necessary before getting a reasonably robust answer, it is difficult to know which meta-analyses will change further and which will not. They conclude “it is still unclear what characteristics of early trials will lead to more confidence being placed in the results of individual meta-analyses” [35].

It is also important to recognise how unreliable initial evidence can be [60]–[61]. An analysis of data from 85,000 meta-analyses with binary outcomes in Cochrane Reviews showed that early trials tend to overestimate treatment effects [62]. This may result, for example, from studies selecting unrepresentative subgroups of patients known to have responded favourably to similar interventions previously, or from excluding patients who have not responded [63]; from biased under-reporting of early trials with disappointing results [64]; or time-lag bias in the publication of the results of trials [65]. As a result, early studies and meta-analyses of them may tend to yield inflated estimates of effects and this needs to be taken into account in considering proposals for additional studies [66]. However, an assessment of the existing evidence is still crucial to providing the ethical, scientific and environmental justification for proposed new trials. Without such reviews, those responsible for proposed new trials cannot make a well-informed decision about whether to proceed with them, and they need to include careful assessment of the quality and risk of bias in the studies being brought together [67]. This should influence the interpretation of the results of the cumulative meta-analysis, alongside consideration of the possibility that spurious results might arise due to cumulative testing. This may require the use of statistical techniques such as sequential analysis, and the need to consider the statement “Don't Ignore Chance Effects” when building from early, positive findings [68].

In considering possible limitations of our research, we note that systematic reviews of healthcare interventions are subject to the impact of selective reporting by researchers, in which whole studies might not be published [69] or, even if studies are published, particular findings might be excluded because of the authors' or editors' opinions about the findings [70]. This methodological review could also be subject to such biases, where cumulative meta-analyses may have been performed but not reported. In contrast to clinical trials [71] or, more recently, systematic reviews with health outcomes [72], there is no widely available system to register prospectively methodological research, such as cumulative meta-analysis. This makes it impossible to determine the extent of selective reporting of cumulative meta-analysis, or its potential impact on our conclusions. Furthermore, although there were relatively few examples of cumulative meta-analyses in which benefits appeared for an intervention after the initial trials had shown null or negative results, this is not surprising, because awareness of such early results, even without a formal meta-analysis, might discourage future research. By contrast, while early positive results might lead to the conduct of new trials to confirm those results or to test their reproducibility in other settings [73].

Despite these limitations, however, and given that this review found such a breadth of examples, it is likely that our general finding is likely to be valid, namely, that there is a substantial problem of waste in research resulting from unnecessary duplication because existing research has not been reviewed before and after new studies are done [4].

## Conclusions

This analysis of 50 reports including over 1500 cumulative meta-analyses of clinical intervention studies shows that, had researchers assessed systematically what was already known, some beneficial and harmful effects of treatments could have been identified earlier and might have prevented the conduct of the new trials. This would have led to the earlier uptake of effective health and social care interventions in practice, less exposure of trial participants to less effective treatments, and reduced waste resulting from unjustified research.

We do not argue that the conduct of a new trial in the presence of apparent certainty from a meta-analysis of existing research is necessarily wrong in all circumstances. The new trial might serve to fill a gap or resolve a remaining uncertainty for particular types of intervention, patient or setting, or seek to assess important outcomes that were not measured in the earlier trials [74]. However, we do argue that people designing, funding, conducting and then interpreting new studies should make their decision to do so in light of an up-to-date systematic review and, if possible, meta-analysis of existing related research. This would allow them to show the ethical, scientific and environmental basis for their proposed new trials, and to demonstrate that they would not be wasteful and justify their decision [8].

## Supporting Information

Appendix S1 **Cumulative meta-analyses of studies of the effects of healthcare interventions.**
(DOCX)
