# Supplementary material for: Accumulating Research: A Systematic Account of How Cumulative Meta-Analyses Would Have Provided Knowledge, Improved Health, Reduced Harm and Saved Resources
Source: PLoS One. 2014 Jul 28;9(7):e102670. doi: 10.1371/journal.pone.0102670 (PMC4113310; doi:10.1371/journal.pone.0102670)
Supplement: Appendix S1 — Cumulative meta-analyses of studies of the effects of healthcare interventions. (DOCX) [file pone.0102670.s001.docx]

**Appendix S1 Cumulative meta-analyses of studies of the effects of healthcare interventions**

Algra A, van Gijn J (1999) Cumulative meta-analysis of aspirin efficacy after cerebral ischaemia of arterial origin. Journal of Neurology, Neurosurgery and Psychiatry 66: 255.

Antman EM, Lau J, Kupelnick B, Mosteller F, Chalmers TC (1992) A comparison of results of meta-analyses of randomized control trials and recommendations of clinical experts. Treatments for myocardial infarction. JAMA 268: 240-248.

Barker FG (1994) Efficacy of prophylactic antibiotics for craniotomy: a meta-analysis. Neurosurgery 35: 484-490.

Belsey J, Crosta C, Epstein O, Fischbach W, Layer P,et al (2012) Meta-analysis: the relative efficacy of oral bowel preparations for colonoscopy 1985-2010. Alimentary Pharmacology & Therapeutics 35: 222-237.

Bhandari M, Schemitsch E, Jonsson A, Zlowodzki M, Haidukewych GJ (2009) Gamma nails revisited: gamma nails versus compression hip screws in the management of intertrochanteric fractures of the hip: a meta-analysis. Journal of Orthopaedic Trauma 23: 460-44.

Bollen CW, Uiterwaal CS, van Vught AJ (2003) Cumulative metaanalysis of high-frequency versus conventional ventilation in premature neonates. American Journal of Respiratory and Critical Care Medicine 168: 1150-1155.

Brok J, Gluud LL, Gluud C (2010) Meta-analysis: ribavirin plus interferon vs. Interferon monotherapy for chronic hepatitis C - an updated Cochrane review. Alimentary Pharmacology and Therapeutics 32: 840-850.

Carpinelli L, Primignani M, Bianchi M, Civettini F, De Franchis R (1995) Treatment of bleeding oesophageal varices: A meta-analysis. Argomenti di Gastroenterologia Clinica 8: 5-15.

Clark O, Adams JR, Bennett CL, Djulbegovic B (2002) Erythropoietin, uncertainty principle and cancer related anaemia. BMC Cancer 2: 23.

Coomarasamy A, Thangaratinam S, Gee H, Khan KS (2006) Progesterone for the prevention of preterm birth: a critical evaluation of evidence. European Journal of Obstetrics, Gynecology, & Reproductive Biology 129: 111-118.

Davey-Smith G, Song F, Sheldon TA (1993) Cholesterol lowering and mortality: the importance of considering initial level of risk. British Medical Journal 306: 1367-1373.

Dent L, Taylor R, Jolly K, Raftery J (2011) "Flogging dead horses": evaluating when have clinical trials achieved sufficiency and stability? A case study in cardiac rehabilitation. Trials 12: 83.

Deshpande G, Rao S, Patole S, Bulsara M (2010) Trial sequential analysis of updated meta-analysis of probiotics for preventing necrotising enterocolitis in preterm VLBW neonates: are the results conclusive? Journal of Paediatrics and Child Health, Conference: 14th Annual Congress of the Perinatal Society of Australia and New Zealand, PSANZ 2010 Wellington New Zealand. 46: 65.

Devereaux PJ, Beattie WS, Choi,PT, Badner NH, Guyatt GH, et al (2005) How strong is the evidence for the use of perioperative beta blockers in non-cardiac surgery? Systematic review and meta-analysis of randomised controlled trials. British Medical Journal 331: 313-316.

Diener MK, Voss S, Jensen K, Buchler MW, Seiler CM (2010) Elective midline laparotomy closure: the INLINE systematic review and meta-analysis. Annals of Surgery 251: 843-856.

Fergusson D, Glass KC, Hutton B, Shapiro S (2005) Randomized controlled trials of aprotinin in cardiac surgery: could clinical equipoise have stopped the bleeding? Clinical Trials 2: 218-229.

Gilbody S, Bower P, Fletcher J, Richards D, Sutton AJ (2006) Collaborative care for depression: a cumulative meta-analysis and review of longer-term outcomes. Archives of Internal Medicine 166: 2314-2321.

Graudal NA, Galloe AM, Garred P (1998) Effects of sodium restriction on blood pressure, renin, aldosterone, catecholamines, cholesterols, and triglyceride: A meta-analysis. JAMA 279: 1383-1391.

Gupta AK, Ryder JE, Johnson AM (2004) Cumulative meta-analysis of systemic antifungal agents for the treatment of onychomycosis. British Journal of Dermatology 150: 537-544.

Hanson RK, Broom I (2005) The utility of cumulative meta-analysis: application to programs for reducing sexual violence. Sexual Abuse: Journal of Research & Treatment 17: 357-373.

Henderson WG, Moritz T, Goldman S, Copeland J, Sethi G (1995) Use of cumulative meta-analysis in the design, monitoring, and final analysis of a clinical trial: a case study. Controlled Clinical Trials 16: 331-341.

Herbison P, Hay-Smith J, Gillespie WJ (2011) Meta-analyses of small numbers of trials often agree with longer-term results. Journal of Clinical Epidemiology 64: 145-153.

Juni P, Nartey L, Reichenbach S, Sterchi R, Dieppe PA, et al (2004) Risk of cardiovascular events and rofecoxib: cumulative meta-analysis. Lancet 364: 2021-2029.

Ker K, Edwards P, Perel P, Shakur H, Roberts I (2012) Effect of tranexamic acid on surgical bleeding: systematic review and cumulative meta-analysis. BMJ 344: e3054.

Keus F, Wetterslev J, Gluud C, Gooszen HG, van Laarhoven CJHM (2010) Trial sequential analyses of meta-analyses of complications in laparoscopic vs. small-incision cholecystectomy: more randomized patients are needed. Journal of Clinical Epidemiology 63: 246-256.

Klein JB, Jacobs RH, Reinecke MA (2007) Cognitive-behavioral therapy for adolescent depression: a meta-analytic investigation of changes in effect-size estimates. Journal of the American Academy of Child and Adolescent Psychiatry 46: 1403-1413.

Lau J, Antman EM, Jimenez-Silva J, Kupelnick B, Mosteller F, et al (1992) Cumulative meta-analysis of therapeutic trials for myocardial infarction. New England Journal of Medicine 327: 248-254.

Lau J, Schmid CH, Chalmers TC (1995) Cumulative meta-analysis of clinical trials builds evidence for exemplary medical care. Journal of Clinical Epidemiology 48: 45-57.

Li LH, Sun TS, Liu Z, Guo YZ, Li SG, et al (2008) Plating versus intramedullary nailing of humeral shaft fractures in adults: a systematic review. Chinese Journal of Evidence-Based Medicine 8: 662-667.

Martel G, Crawford A, Barkun JS, Boushey RP, Ramsay CR, et al (2012) Expert opinion on laparoscopic surgery for colorectal cancer parallels evidence from a cumulative meta-analysis of randomized controlled trials. PLoS ONE 7: e35292.

Matchaba P, Gitton X, Krammer G, Ehrsam E, Sloan VS, et al (2005) Cardiovascular safety of lumiracoxib: a meta-analysis of all randomized controlled trials ≥1 week and up to 1 year in duration of patients with osteoarthritis and rheumatoid arthritis. Clinical Therapeutics 27: 1196-1214.

Mills EJ, Wu P, Alberton M, Kanters S, Lanas A, et al (2012) Low-dose aspirin and cancer mortality: a meta-analysis of randomized trials. American Journal of Medicine 125: 560-567.

Mittendorf R, Aronson MP, Berry RE, Williams MA, Kupelnick B, et al (1993) Avoiding serious infections associated with abdominal hysterectomy: a meta-analysis of antibiotic prophylaxis. American Journal of Obstetrics and Gynecology 169: 1119-1124.

Moles DR, Needleman IG, Niederman R, Lau J (2005) Introduction to cumulative meta-analysis in dentistry: lessons learned from undertaking a cumulative meta-analysis in periodontology. Journal of Dental Research 84: 345-349.

Oh SW, Myung SK, Park JY, Lym YL, Ju W (2010) Hormone therapy and risk of lung cancer: a meta-analysis. Journal of Women's Health 19: 279-288.

Palmer S, Navaneethan S, Craig J, Johnson D, Tonelli M, et al (2010) Erythropoiesis-stimulating agents in people with chronic kidney disease: a cumulative meta-analysis. NDT Plus, Conference: 17th ERA-EDTA Congress - II DGfN Congress Munich Germany. 3: iii80-iii81.

Peter JV, Moran JL, Phillips-Hughes J, Warn D (2002) Noninvasive ventilation in acute respiratory failure - a meta-analysis update. Critical Care Medicine 30: 555-562.

Poolman RW, Farrokhyar F, Bhandari M (2007) Hamstring tendon autograft better than bone patellar-tendon bone autograft in ACL reconstruction: a cumulative meta-analysis and clinically relevant sensitivity analysis applied to a previously published analysis. Acta Orthopaedica 78: 350-354.

Puhan MA, Vollenweider D, Steurer J, Bossuyt PM, Ter Riet G (2008) Where is the supporting evidence for treating mild to moderate chronic obstructive pulmonary disease exacerbations with antibiotics? A systematic review. BMC Medicine 6: 28.

Ross JS, Madigan D, Hill KP, Egilman DS, Wang Y, et al (2009) Pooled analysis of rofecoxib placebo-controlled clinical trial data: lessons for postmarket pharmaceutical safety surveillance. Archives of Internal Medicine 169: 1976-1985.

Sheth U, Simunovic N, Tornetta P 3rd, Einhorn TA, Bhandari M (2011) Poor citation of prior evidence in hip fracture trials. Journal of Bone and Joint Surgery - Series A 93: 2079-2086.

Sinclair JC (1995) Meta-analysis of randomized controlled trials of antenatal corticosteroid for the prevention of respiratory distress syndrome: discussion. American Journal of Obstetrics and Gynecology 173: 335-344.

Thome U, Carlo WA, Pohlandt F (2005) Ventilation strategies and outcome in randomised trials of high frequency ventilation. Archives of Disease in Childhood: Fetal and Neonatal Edition 90: F466-F473.

Wallace AE, Neily J, Weeks WB, Friedman MJ (2006) A cumulative meta-analysis of selective serotonin reuptake inhibitors in pediatric depression: did unpublished studies influence the efficacy/safety debate? Journal of Child and Adolescent Psychopharmacology 16: 37-58.

Wardlaw JM, Sandercock PAG, Berge E (2003) Thrombolytic therapy with recombinant tissue plasminogen activator for acute ischemic stroke: where do we go from here? A cumulative meta-analysis. Stroke 34: 1437-1442.

Weigel R, Schmiedek P, Krauss JK (2003) Outcome of contemporary surgery for chronic subdural haematoma: evidence based review. Journal of Neurology, Neurosurgery & Psychiatry 74: 937-943.

Wells G, Parkash R, Healey JS, Talajic M, Arnold JM, et al (2011) Cardiac resynchronization therapy: a meta-analysis of randomized controlled trials. Canadian Medical Association Journal 183: 421-429.

Whiting GW, Lau J, Kupelnick B, Chalmers TC (1995) Trends in inflammatory bowel disease therapy: a meta-analytic approach. Canadian Journal of Gastroenterology 9: 405-411.

Zhang J, Ding EL, Song Y (2006) Adverse effects of cyclooxygenase 2 inhibitors on renal and arrhythmia events: meta-analysis of randomized trials. JAMA 296: 1619-1632.

Zhang W, Nuki G, Moskowitz RW, Abramson S, Altman RD, et al (2010) OARSI recommendations for the management of hip and knee osteoarthritis: Part iii: changes in evidence following systematic cumulative update of research published through January 2009. Osteoarthritis and Cartilage 18: 476-499.
